# The epitome of tailor-made short positively charged peptides against HCC via integrated pharmacology

**DOI:** 10.1186/s12967-024-05087-w

**Published:** 2024-03-15

**Authors:** Ki-Kwang Oh, Jung-A Eom, Kyeong Jin Lee, Goo-Hyun Kwon, Sang-Jun Yoon, Seol Hee Song, Jeong Ha Park, Jeong Su Kim, Dong Joon Kim, Ki-Tae Suk

**Affiliations:** Institute for Liver and Digestive Diseases, College of Medicine, Hallym University, Chuncheon, 24252 Korea

**Dear Editor,**

Recently, short positively charged peptides (SPCPs ≤ 500 Dalton) with positively charged residues (Arginine; R, Histidine; H, and Lysine; K) have been identified as alternative therapy with anti-cancer efficacy, which can penetrate plasma membrane to reach at target(s) [1]. The peptides are exposed to unfavorable pharmacokinetic properties such as hydrophilicity, high degradability, and short half-life in oral formulation. Alternatively, intra-tumoral injection (ITUI) is a promising strategy to improve the immunogenicity for anti-tumors [2].

Moreover, cancer cell membrane (CCM) is surrounded by phosphatidylserine (PS) with negatively charged ions in pH 6.2–6.9 [3]. The SPCPs can destroy the CCM with negatively charged residues, which is optimal to develop suitable agents. Thus, SPCPs obtained by R Package filtered to select significant SPCPs with stable physicochemical properties via the reliable web-based bioinformatics tools. The layout of this study was depicted in Fig. 1A.

**Fig. 1:**
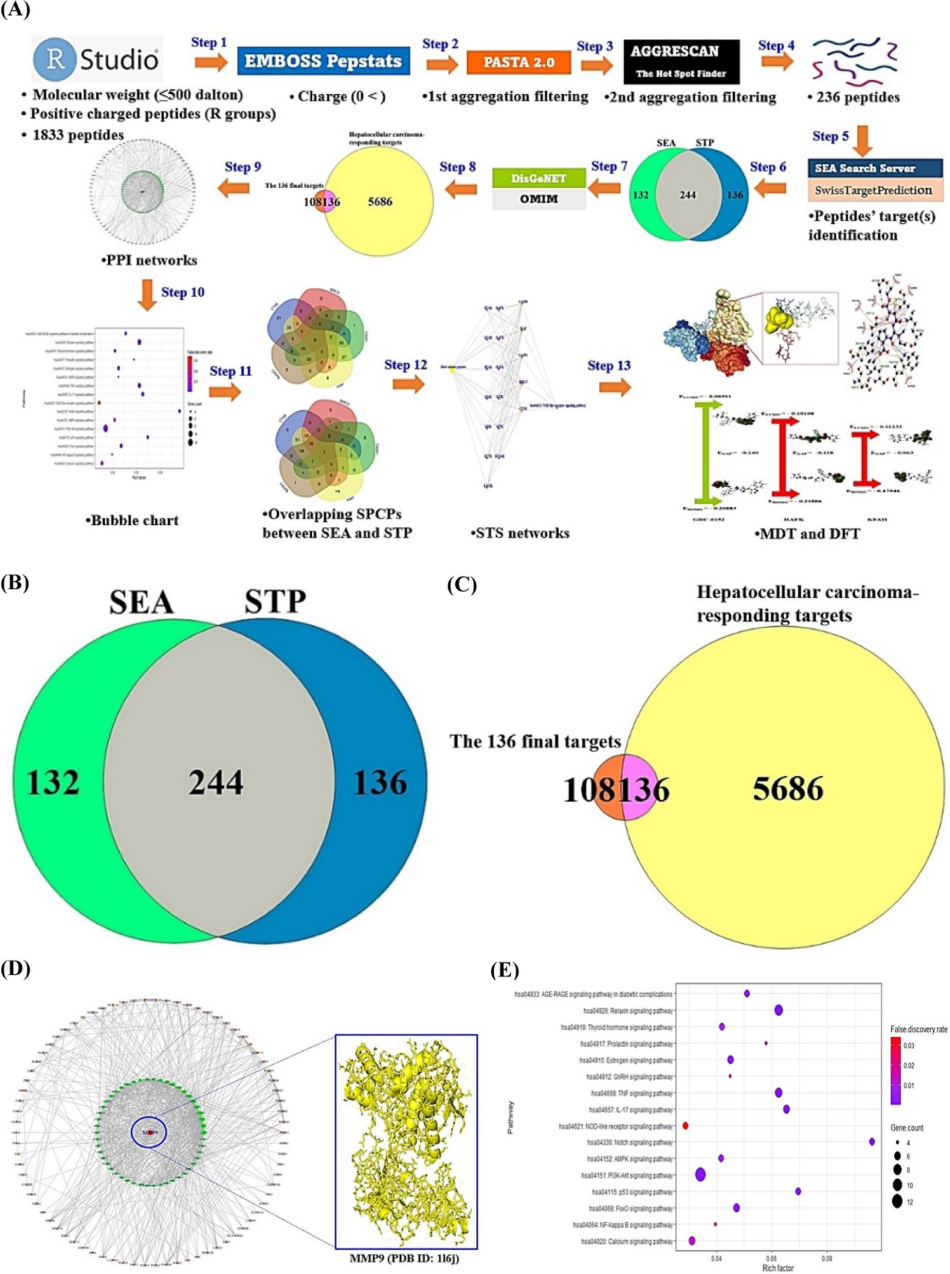

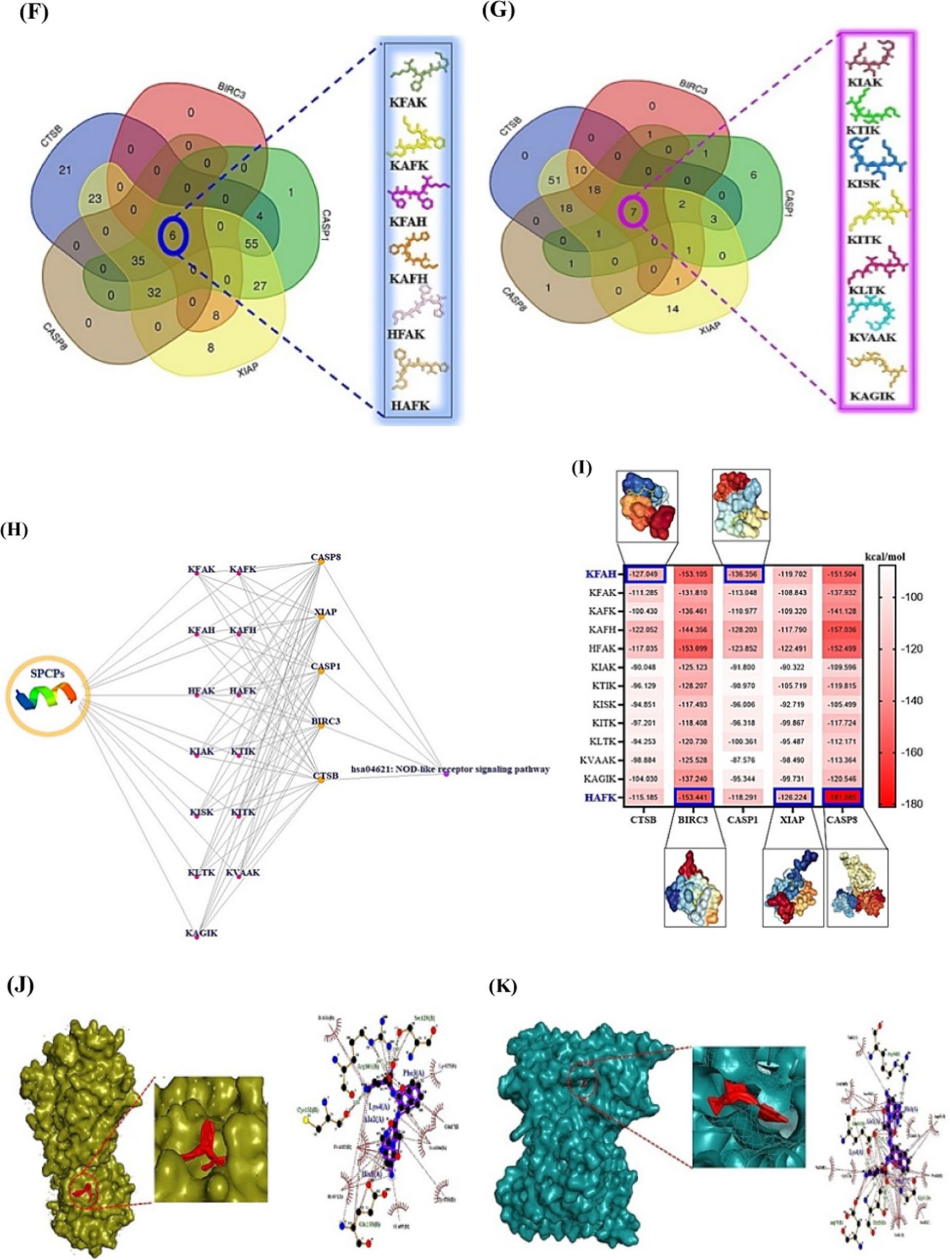

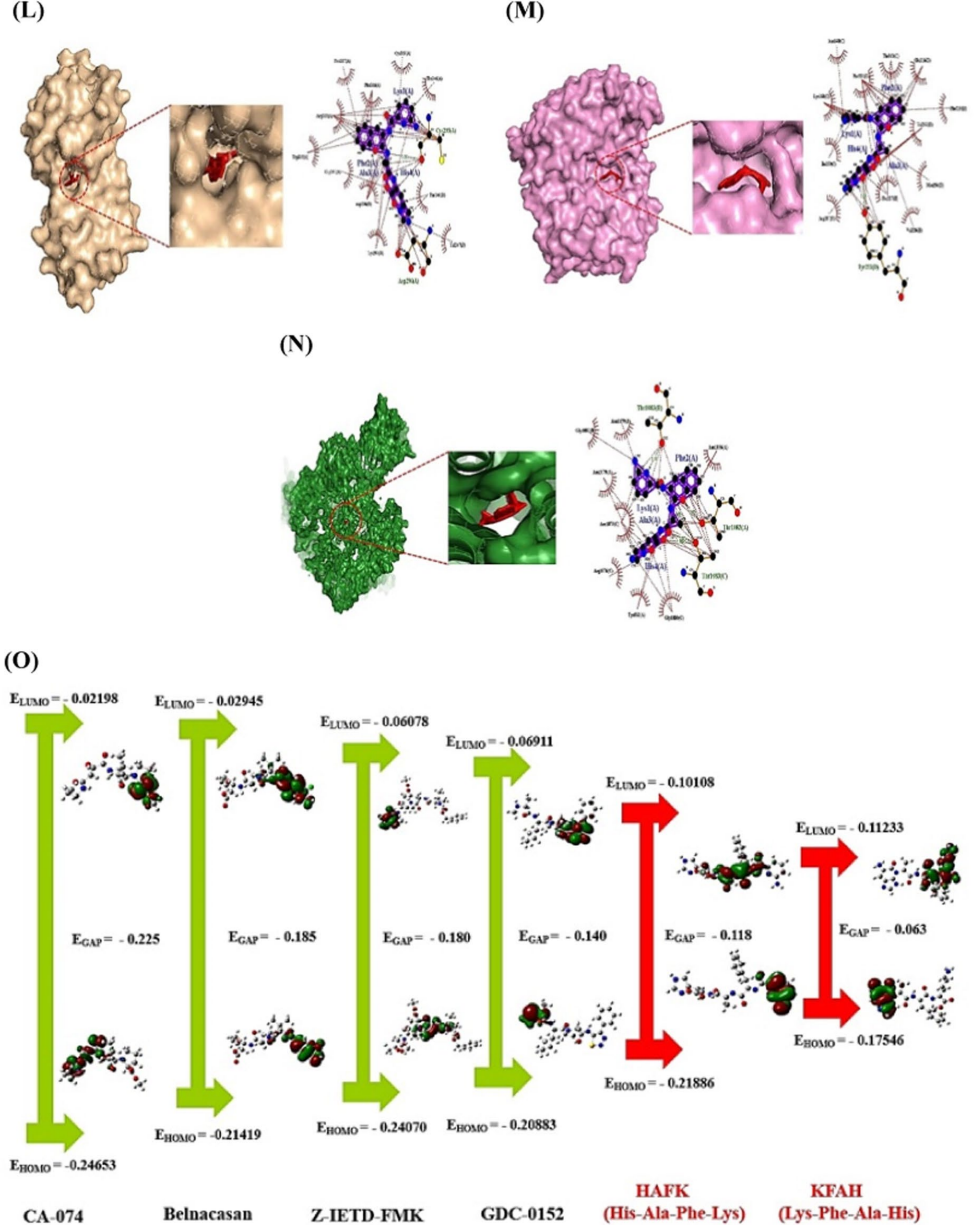
**A** The mining workflow of short positively charged peptides (SPCPs) of this study. **B** The number of 244 targets between SEA and STP. **C** The final 136 targets for the analysis. **D** The protein–protein interaction (PPI) networks. **E** The 16 signaling pathways related to hepatocellular carcinoma. **F** The number of 6 intersecting SPCPs in 5 key targets from SEA. **G** The number of 7 intersecting SPCPs in 5 key targets from STP. **H** The SPCPs-targets-signaling pathway (STS) networks. A: Alanine; F: Phenylalanine; H: Histidine; K: Lysine; S: Serine; T: Threonine; V: Valine. **I** The docking scores on 2 SPCPs (KFAH, and HFAK)-5 key targets (CTSB, CASP1, BIRC3, XIAP, and CASP8) via HPEPDOCK 2.0. Blue rectangles: the most significant conformers on SPCPs-key targets. **J** The CA-074—CTSB conformer via AutoDock. **K** The Belnacasan—CASP1 conformer via AutoDock. **L** The GDC-0152—BIRC3 conformer via AutoDock. **M** The GDC-0152—XIAP conformer via AutoDock. **N** The Z-IETD-FMK—CASP8 conformer via AutoDock. **O** The comparison of energy gap between two key SPCPs and standard compounds

Firstly, the definition SPCPs is as below.

1. Peptide(s) less than 500 Dalton (DA).
2. Peptides with cationic amino acids on N-terminal, and C-terminal.

The 1833 SPCPs (Additional file 1: Table S1) to be accepted by the two criteria were input to EMBOSS Pepstats (https://www.ebi.ac.uk/Tools/seqstats/emboss_pepstats/) to obtain the SPCPs with specific isoelectric point between 8 and 12. Then, SPCPs with non-aggregated propensity were filtered in PASTA 2.0 (https://protein.bio.unipd.it/), and AGGRESCAN (− 40 ≤ Na4VSS ≤ 60) (http://bioinf.uab.es/aggrescan/). The 236 out of 1833 SPCPs was accepted by the filtering point (Additional file 1: Table S1).

Secondly, the targets on SPCPs were retrieved by Similarity Ensemble Approach (376 targets) and SwissTargetPrediction (380 targets), indicating that the overlapping targets (244 targets) might be significant elements in SPCPs (Additional file 2: Table S2) (Fig. 1B). The HCC-responding targets (5822 targets) were identified by DisGeNET and OMIM databases, compared with the 244 targets (Additional file 2: Table S2). The 136 targets were considered as therapeutic targets against HCC via SPCPs (Additional file 2: Table S2) (Fig. 1C).

Thirdly, the final 136 targets were put into STRING platform to represent the protein–protein interaction (PPI) networks, thereby, 129 targets were interconnected directly to each one another (129 nodes, and 800 edges) (Fig. 1D). The highest-ranking target in the PPI networks was MMP9 with the greatest degree of value (46) (Additional file 3: Table S3). The Kyoto Encyclopedia of Genes and Genomes (KEGG) pathway enrichment analysis was represented 16 signaling pathways associated with the occurrence and progression of HCC (Fig. 1E) (Additional file 4: Table S4). It implied that NOD-like receptor signaling pathway with the lowest rich factor might be an antagonism to alleviate HCC. The 5 targets (BIRC3, CASP1, CASP8, CTSB, and XIAP) related to NOD-like receptor signaling pathway were considered as protein coding genes to combat HCC. More importantly, the 6 SPCPs has been intersected with the 5 targets, suggesting that the SPCPs might be significant effector(s) (Additional file 5: Table S5), (Fig. 1F). Likewise, the 7 SPCPs has been overlapped with the 5 targets, indicating that the SPCPs might be important agent(s) (Additional file 5: Table S5), (Fig. 1G). The SPCPs-targets-signaling pathway networks consist of 20 nodes and 83 edges, representing that the three components might function as therapeutic module (Fig. 1H).

Lastly, in the molecular docking test, the two SPCPs (KFAH, and HFAK) formed the most stable complex on the 5 key targets (Fig. 1I). The KFAH had the strongest affinity on CTSB, and CASP1, the HAFK bound most stably to BIRC3, XIAP, and CASP8. The AutoDock program was employed to confirm its affinity with rigorousness by comparison with standard drugs (Additional file 6: Table S6) (Fig. 1J–N). As a result, the two key SPCPs had more potent affinity than known ligands. Additionally, density functional theory (DFT) analysis was performed to clarify the aptness of the physicochemical properties (Additional file 7: Table S7). The two SPCPs manifested that they have high chemical reactivity (Fig. 1O) and conformer stability.

In conclusion, the pinpointed SPCPs (KFAH, and HAFK) had more potency than existing drugs for the treatment of HCC, the mechanism of which was to dampen NOD-like receptor signaling pathway on the 5 targets. Thereby, the two key SPCPs are capable to exert to antagonism, supporting that they can be developed into ITUI formulation to evade the pharmacokinetic drawbacks in systemic circulation. The key summary of this study was unfolded in Fig. 2.

**Fig. 2:**
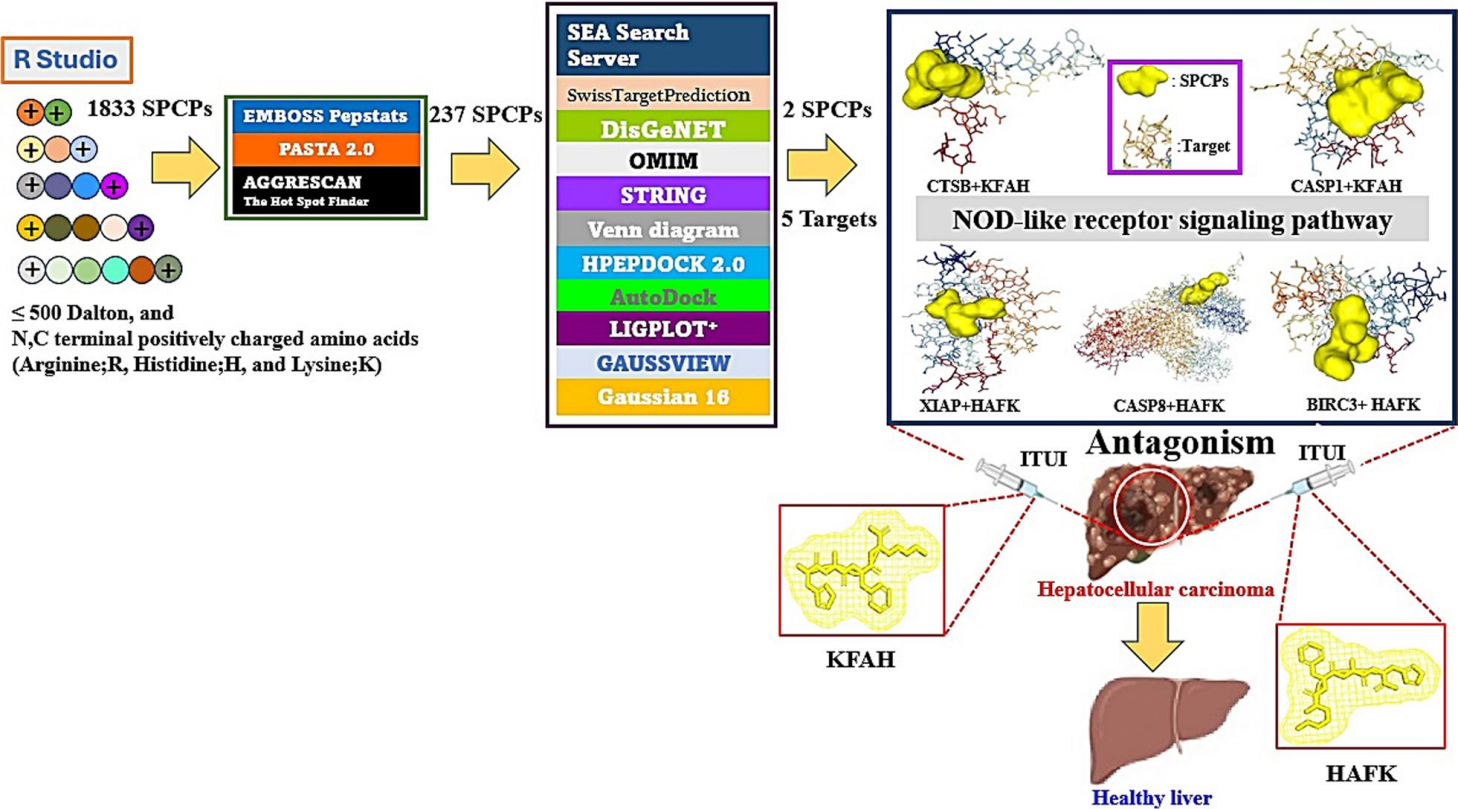
The key summary of this study

### Supplementary Information

**Additional file 1: Table S1.** The profiling of short positively charged peptides (SPCPs).

**Additional file 2: Table S2.** The profiling of key targets in this study.

**Additional file 3: Table S3.** The degree of value on targets.

**Additional file 4: Table S4.** The description of 16 signaling pathways related to occurrence and progression of HCC.

**Additional file 5: Table S5.** The list of potential SPCPs on NOD-like receptor signaling pathway.

**Additional file 6: Table S6.** The docking score of key SPCPs and standard compounds.

**Additional file 7: Table S7.** The DFT parameters of two key SPCPs and three standard drugs.

## Data Availability

All data generated or analyzed during this study are included in this published article (and its Additional files).

## Abbreviations

R: Arginine
CCM: Cancer cell membrane
DA: Dalton
DFT: Density functional theory
χ: Electronegativity
E_GAP_: Energy gap
FMO: Frontier molecular orbitals
ɳ: Hardness
HCC: Hepatocellular carcinoma
HAFK: His-Ala-Phe-Lys
H: Histidine
ITUI: Intra-tumoral injection
KEGG: Kyoto Encyclopedia of Genes and Genomes
K: Lysine
KFAH: Lys-Phe-Ala-His
PS: PhosphatidylSerine
PPI: Protein–protein interaction
SPCP: Short positively charged peptide
SPCPs: Short positively charged peptides
S: Softness

## References

[1] Bitler BG, Schroeder JA (2010). Anti-cancer therapies that utilize cell penetrating peptides. Recent patents on anti-cancer drug discovery. Recent Pat Anticancer Drug Discov.

[2] Nobuoka D, Yoshikawa T, Takahashi M, Iwama T, Horie K, Shimomura M (2013). Intratumoral peptide injection enhances tumor cell antigenicity recognized by cytotoxic T lymphocytes: a potential option for improvement in antigen-specific cancer immunotherapy. Cancer Immunol Immunother.

[3] Alves AC, Ribeiro D, Nunes C, Reis S (2016). Biophysics in cancer: the relevance of drug-membrane interaction studies. Biochimica et Biophysica Acta BBA Biomembranes..

